# Supplementary figures and images for: Influence of long term nitrogen limitation on lipid, protein and pigment production of Euglena gracilis in photoheterotrophic cultures
Source: PeerJ. 2019 Apr 1;7:e6624. doi: 10.7717/peerj.6624 (PMC6448558; doi:10.7717/peerj.6624)

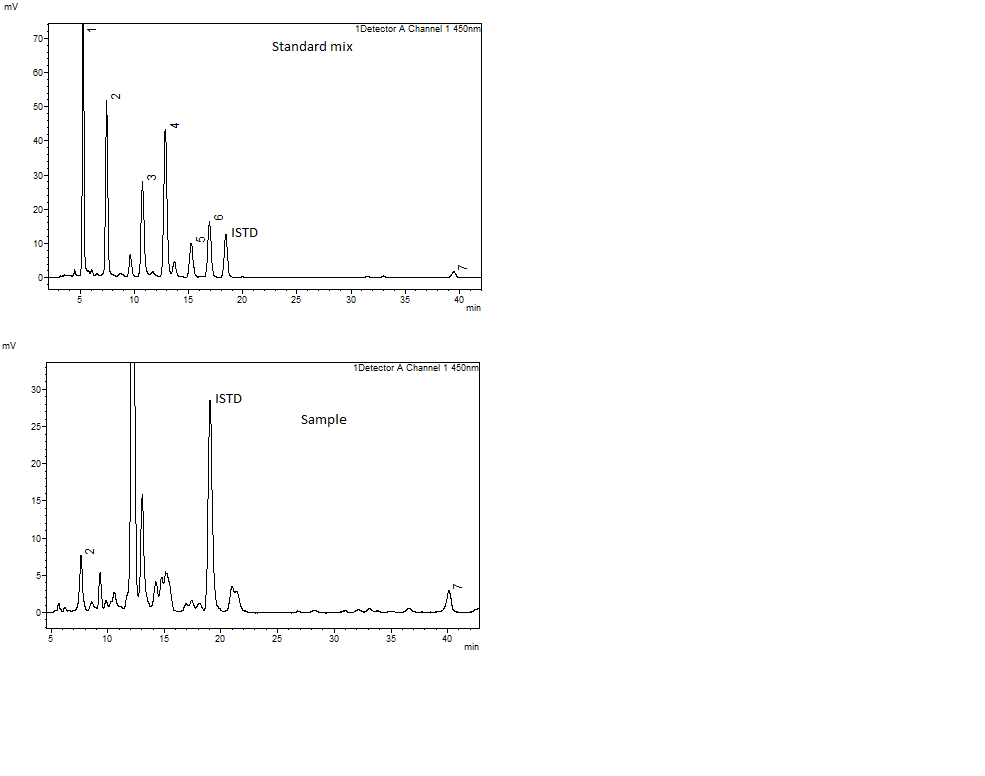

Supplement: Supplemental Information 1 — Peaks and retention times in standard mix. 1. fucoxanthin (rt = 5.25), 2. neoxanthin (rt = 7.42), 3. astaxanthin (rt = 10.73), 4. zeaxanthin (rt = 15.24), 5. cantaxanthin (16.92), and 6. β-carotene (39.49). Corresponding peaks in sample are shown. [file peerj-07-6624-s001.png]

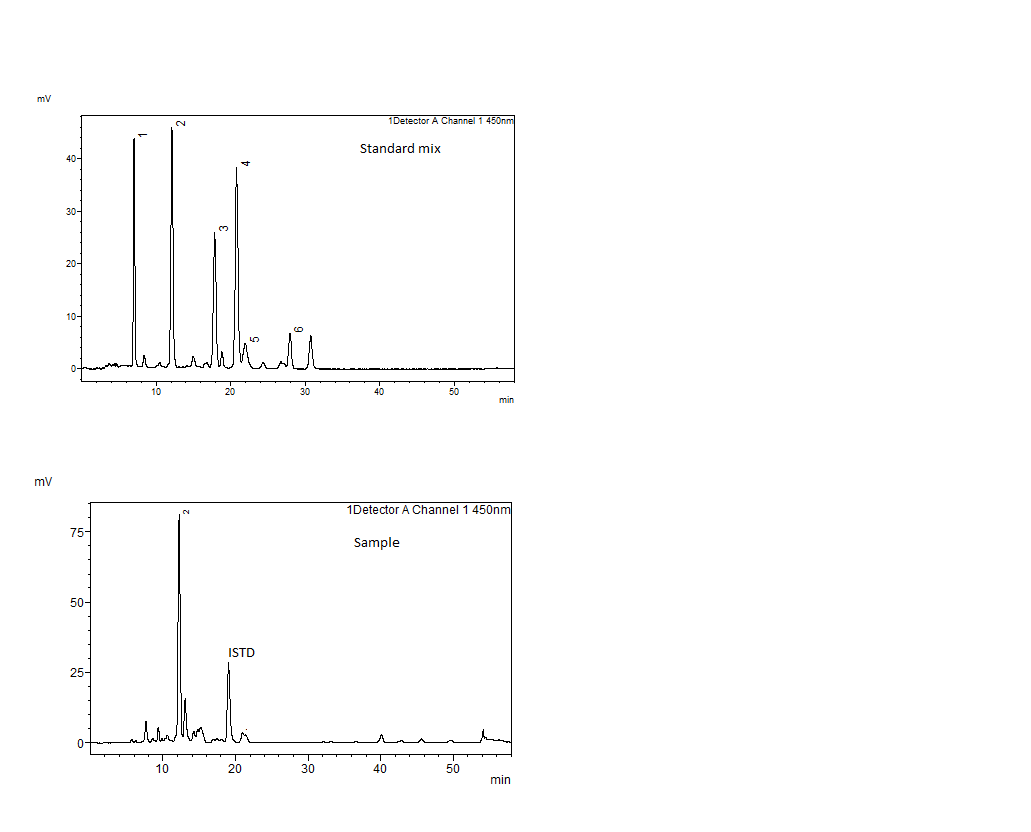

Supplement: Supplemental Information 2 — Peaks and retention times in standard mix. 1. violaxanthin (rt = 7.06), 2. diadinoxanthin (rt = 12.12), 3. diatoxanthin (17.86), 4. alloxanthin (rt = 20.80), 5. myxoxantophyll (rt = 21.93), and 6. echinenone (rt = 27.95). Corresponding peaks in sample are shown. [file peerj-07-6624-s002.png]
